# Automatic identification of tinnitus malingering based on overt and covert behavioral responses during psychoacoustic testing

**DOI:** 10.1038/s41746-022-00675-w

**Published:** 2022-08-29

**Authors:** Christopher J. Smalt, Jenna A. Sugai, Elouise A. Koops, Kelly N. Jahn, Kenneth E. Hancock, Daniel B. Polley

**Affiliations:** 1 Human Health & Performance Systems Group, MIT Lincoln Laboratory, Lexington, MA USA; 2 Eaton-Peabody Laboratories, Mass Eye and Ear, Boston, MA USA; 3 Department of Neuroscience, University of Groningen, Groningen, Netherlands; 4 Department of Otolaryngology - Head and Neck Surgery, Harvard Medical School, Boston, MA USA

**Keywords:** Cortex, Diagnosis

## Abstract

Tinnitus, or ringing in the ears, is a prevalent condition that imposes a substantial health and financial burden on the patient and to society. The diagnosis of tinnitus, like pain, relies on patient self-report, which can complicate the distinction between actual and fraudulent claims. Here, we combined tablet-based self-directed hearing assessments with neural network classifiers to automatically differentiate participants with tinnitus (*N* = 24) from a malingering cohort, who were instructed to feign an imagined tinnitus percept (*N* = 28). We identified clear differences between the groups, both in their overt reporting of tinnitus features, but also covert differences in their fingertip movement trajectories on the tablet surface as they performed the reporting assay. Using only 10 min of data, we achieved 81% accuracy classifying patients and malingerers (ROC AUC = 0.88) with leave-one-out cross validation. Quantitative, automated measurements of tinnitus salience could improve clinical outcome assays and more accurately determine tinnitus incidence.

## Introduction

Tinnitus is a prevalent sensory disorder that imposes a substantial burden to the patient and to society^1,2^. According to the US veteran benefits administration fiscal year 2020, tinnitus was the most prevalent service-connected disability among new compensation recipients. In fiscal year 2020, 10.2% of all new benefits recipients received compensation for tinnitus (149,368 claims). Tinnitus was also the most prevalent overall disability with a total of 2,327,387 claims of tinnitus that year, with hearing loss as the second most prevalent disability claimed, representing 1,343,013 claims. The economic and health impacts of tinnitus are alarming not only because of the high prevalence, but also because tinnitus claims have been consistently rising at an average annual rate of 12% since 2011. It is noteworthy that since that time, tinnitus claims have been growing at nearly twice the rate of hearing loss (7%), a related condition for which there is an established diagnostic measurement via the pure tone audiogram.

Currently, the diagnosis of tinnitus, like pain, relies on subjective and self-reported measures^3^. As a result, it is challenging for patients to convey the characteristics and severity of their tinnitus percept to their caregivers. Although psychoacoustic measures such as tinnitus pitch and intensity matching are often obtained to define the auditory characteristics of tinnitus, there is presently no established relationship between these attributes and the actual severity of the symptom^4–6^. Both the quality and severity of tinnitus can vary over time for an individual^7,8^. Test-retest reliability across patients has been shown to be as high as 0.94 on a 52-item questionnaire^9^, but much poorer across longer time spans of months^10^ reflecting the dynamic nature of tinnitus.

Developing new automated and quantitative tinnitus diagnostics could prove useful for assessing tinnitus severity and for distinguishing been legitimate and fraudulent cases among the large—and ever growing—tinnitus disability claims. In this context, malingering is the feigning of a medical condition for gain. Possible incentives for doing so include obtaining economic compensation or to be removed from difficult circumstances, such as military service^11^ or for early retirement. Previous work has shown mixed results in distinguishing malingerers from tinnitus patients using psychoacoustic measures^12,13^, which may reflect the inherently fluctuant nature of tinnitus and the associated challenges this introduces for test-retest reliability for any given tinnitus subject over time^8,14^.

In this study, we develop a system that automatically classifies (i.e. “identifies”) tinnitus patients from malingerers through self-directed, computerized behavioral testing that that asks subjects to characterize the perceptual qualities of their tinnitus. Automatic diagnostic tools are becoming increasingly common across the medical field, driven largely by the success of machine learning (i.e. artificial intelligence) techniques applied to fields including medical imaging^15^, and general image classification^16^ and automatic speech recognition^17^. To predict whether a given subject has tinnitus or was instructed to malinger, we employ binary classification, a type of machine learning, and contrast that approach with more traditional statistical hypothesis testing^18^. First, we analyze the performance of logistic regression and random-forest classification algorithms applied to manually-derived behavioral features (diagnostic measures)^19^. Then, we employ a convolutional deep neural network (DNN) binary classifier with the behavioral measurements in their most raw form, the fingertip movement trajectories. Finally, we compare the aforementioned machine learning techniques and suggest a path towards a deployable, remote diagnostic aid to determine whether an individual has tinnitus.

## Results

### Tinnitus characterization

**Fig. 1:**
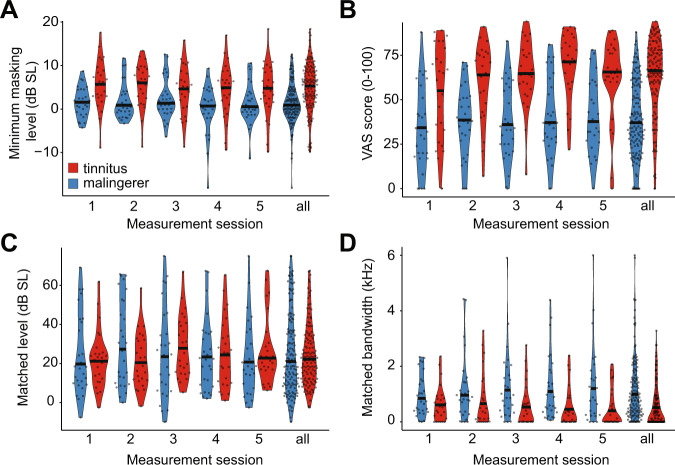
Mean behavioral tinnitus characterization for the patient and malingering group. Normalized density distributions along with single participant data (filled circles) are provided for MML (**A**), VAS (**B**), loudness matching (**C**) and bandwidth matching (**D**). All estimates were repeated five times, shown individually and collapsed over all sessions. Black lines indicate the mean score, and black dots correspond to individual participants. In our population, malingering participants report that their imagined tinnitus is less tonal, more easily masked, and less bothersome than participants with tinnitus.

Patients and malingerers quantified their perceived severity and psychoacoustic qualities of their (imagined) tinnitus in several ways, including visual analog scale rating, minimum masking level (MML), and tinnitus acoustic matching. Figure 1 illustrates the MML (A), the visual analog scale (VAS) tinnitus rating (B), as well as the sound level (C) and spectral bandwidth (D) that matches the (imagined) tinnitus sound. At a group level, when compared to patients with tinnitus, malingerers reported that their tinnitus was masked at lower sound levels, was less bothersome, and had broader spectral bandwidth (i.e., less like a pure tone) (Repeated-measures ANOVA main effect for group on MML (*F* = 9.28, *p* = 0.004), VAS rating (*F* = 31.7, *p* < 0.0001), matching bandwidth (*F* = 7.31, *p* = 0.009). No differences were noted in the sound level used to match the tinnitus loudness between groups (*F* = 0.005, *p* = 0.94). No significant effect of experimental session or interaction effect was observed.

### Classification based on outcome measures

**Fig. 2:**
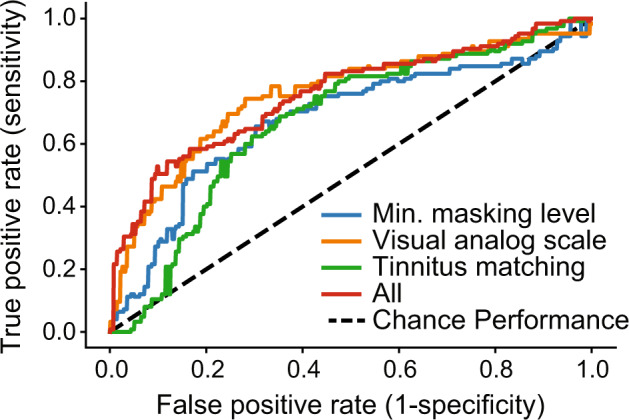
Receiver operator characteristic linear regression on tinnitus psychoacoustic task outcomes. Combining the outcomes across MML, VAS, and Matching tasks achieved a 66% Accuracy and an AUC = 0.75.

While pyschoacoustic and self-reported tinnitus measures indicate statistical differences between the tinnitus and malinger groups, our motivation for the study was to determine if tinnitus status could be determined automatically on an individual level. Figure 2 shows how accurately logistic regression models were able to classify patients from malingerers considering only the final value of each slider (the outcome variable(s)) for each task as the predictor. The MML and VAS classifiers were trained using a single outcome variable corresponding to individual sliders, while the classifier for the matching task had four corresponding to the four sliders used. Finally, the performance combining all slider outcomes is shown in red, achieving 68% Accuracy and an AUC = 0.75.

### Slider time-series

**Fig. 3:**
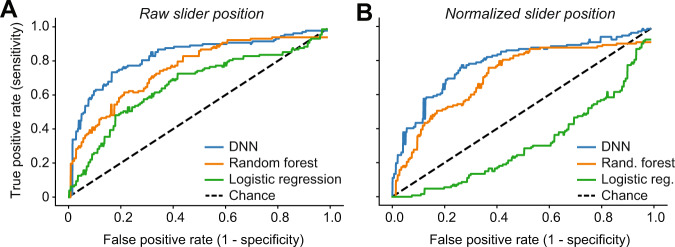
Tinnitus vs malingerer classifier performance using minimum masking level (MML) slider time-series data. **A** The original time-series data with ROC AUC = 0.84, 0.77, and 0.67 and accuracies of 77%, 65%, 49% accuracy respectively for a DNN, Random Forest, and Logistic Regression. **B** The same classifiers on time-series data where the MML value is subtracted out for each participant with an ROC AUC = 0.79, 0.72, and 0.31 and 77.4%, 65%, 49% accuracy respectively.

**Fig. 4:**
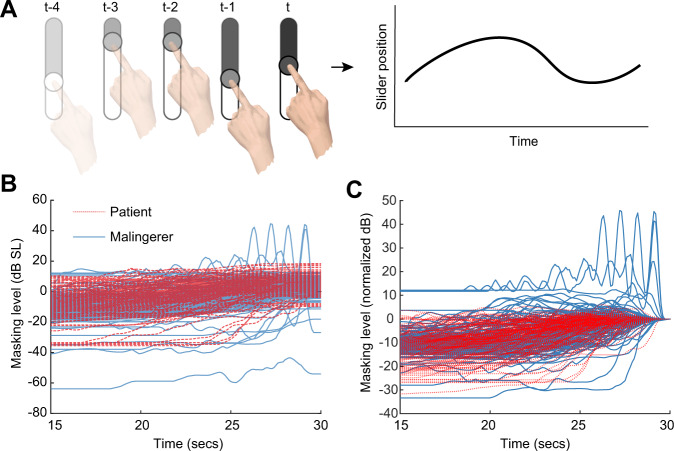
Minimum masking level (MML) slider position as a function of time for tinnitus patients and malingerers. Only the last 15 s of the track were extracted for further analysis. **A** Time history of the slider interaction are collected and stored. **B** Raw finger slider position for the MML task. **C** Normalized finger slider position for the MML task.

In addition to classifying patients from malingers based on the final outcome variables (the final placement of each individual slider), we also created a model to classify the two groups based on engagement of the participant with the sliders during the entirety of the MML task (i.e., how they responded, rather than what they responded). Figure 3A compares the approach using only the pyschoacoustic outcome variable (logistic regression, green) with two alternative classification approaches using the additional features derived from the slider time-series. The feature-based random forest (orange) and DNN (blue) utilize the raw slider time series shown in Fig. 4C. The respective performances were AUC = 0.67, 0.77, and 0.84 with corresponding accuracies of 49%, 65%, and 77%. The Random Forest, which incorporates features such as participant variability within a session, outperforms the classifier relying on the outcome variable alone (logistic regression). The DNN outperforms both with the highest performance, likely due to the access to raw data and potential latent features in the slider time-series data.

A close inspection of the finger path trajectories on the MML task confirmed that tinnitus participants interfaced with the virtual slider differently than malingering participants. As illustrated in (Fig. 1), tinnitus participants gradually increased the making level to converge on their MML, whereas malingering participants made larger, more erratic adjustments before abruptly stopping on their MML. To analyze the degree that the DNN relied on the slider time-series versus the outcome variable (MML), we subtracted the MML for each subject from each time-series (Fig. 4D). This normalization resulted in the respective ROC: AUC = 0.79, 0.72, and 0.31, and accuracies of 77.4%, 65%, 49% for the DNN, random forest, and logistic regression, respectively (Fig. 3B). As expected, the logistic regression performed at chance, since it only used the outcome variable that had been normalized away on average. However, the other two methods show only a slight dip in performance, indicating that the manner in which the slider was adjusted, i.e., how malingering subjects adjust the slider over time rather than the final psychoacoustic measurement value, can be used to discriminate the two populations.

### Feature-based classification

**Fig. 5:**
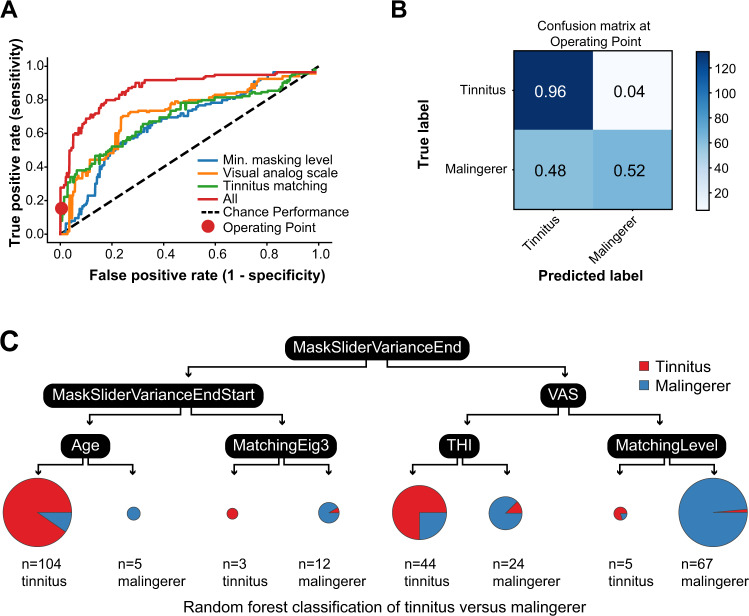
Machine learning performance for binary classification of tinnitus vs. malingerers across behavioral tasks. **A** ROC for tinnitus versus malingerer classification on three psychoacoustic tasks (blue, green, yellow), and combined for all tasks (red) using a random forest. Combined performance accuracy was 81% (AUC = 0.88). **B** Confusion matrix for an operating point with a < 5% false alarm rate. **C** Example decision tree from the random forest classifier in panel (**A**), combining all features.

Finally, we considered a feature-based approach that captured both the outcome variable and slider interaction across all three tasks (VAS, MML, Matching) while maintaining interpretability (as opposed to a Neural Network). ROC curves are shown on each task separately and combined in a single system (Fig. 5A). The MML, VAS, and Matching tasks separately achieved AUCs of 0.68, 0.72, and 0.70 and accuracies of 65%, 70%, 66%, respectively. The combined system performance additionally included other demographic information, such as age and self-reported sex, as well as the subjective tinnitus handicap inventory (THI) score. The combined system performance achieved 81% accuracy with an ROC of 0.88, outperforming any individual task. Figure 5B reports confusions for a system at the operating point shown as red circle in panel A. We selected false-alarm rate of 4%, resulting in a hit rate of 96%. This means that a patient with tinnitus will be identified as such 96% of the time, and 4% of the time they would be incorrectly classified as a malingerer (false-alarm). While this system configuration results in an overall system poorer performance of 75% accuracy, limiting false-alarms is critical if such a system were used to aid health benefit decisions.

Figure 5C illustrates a single decision tree from the random forest derived from features across all three tasks. In this example tree, the root node feature is the standard deviation across trials of the MML slider end point (MaskingSliderStdTrackEnd). Typically, the root node feature is best at separating the two populations, while other features further down in the tree are important but of decreasing use in the separation; in this example, these features include the VAS slider value, the subject age and THI. An advantage to this type of classifier is in its interpretability for medical diagnoses or financial decisions.

**Fig. 6:**
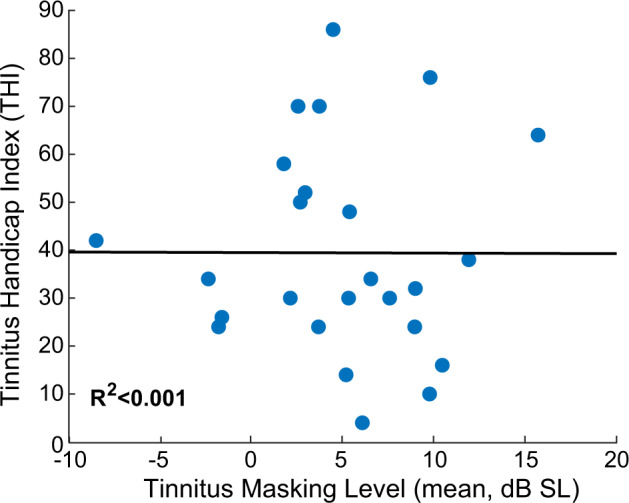
Tinnitus handicap index (THI) score versus minimum masking level (MML). Minimal correlation is observed between a patients reported tinnitus severity and the sound level required to mask their tinnitus.

An important aim for automated tinnitus classification approaches would be to not only address whether subjects hear a phantom sound, but to determine the psychological burden that their tinnitus imposes on their lifestyle. The psychoacoustic characterizations employed here are designed to characterize the perceptual qualities of tinnitus and can be useful in determining whether or not subjects perceive tinnitus. However, perceptual descriptors of tinnitus are known to be poorly correlated with the psychological burden imposed by tinnitus, as captured by widely used questionnaires^4–6^. Based on their THI score, subjects from our tinnitus cohort reported lifestyle burdens ranging from slight (an index of 0–16) to catastrophic (>77), but, in agreement with previous studies, their THI score had no association with how easily their tinnitus was masked by broadband sound (*R*^2^ < 0.001, Fig. 6). As psychological burden is a key attribute of tinnitus, both as a clinical outcome and as a feature that can be feigned for personal gain, expanding the objective classification approach described here to include behavioral measures more indicative of psycho-emotional distress would be an important future direction.

## Discussion

This study aimed to distinguish participants with tinnitus from participants feigning tinnitus using their responses on several standard tinnitus characterization tests. Our results demonstrate that it is possible to discriminate individuals with tinnitus from malingerers with an accuracy of 81% using approximately 10 min worth of reporting data. On average, tinnitus patients reported a higher MML relative to their audiometric threshold, and higher loudness on a visual analog scale and through tinnitus matching. By contrast to prior reports, we did not observe that malingering participants matched their imagined tinnitus to higher physical sound levels than participants with tinnitus^6^.

However, in this study we went beyond characterizing the group differences and extracted information about how the participant interacts with their tablet as opposed to only utilizing their psychoacoustic test results. Our analysis revealed that information about the populations is contained in both how they interacted with the slider as well as in the final slider value. This result has implications for many other fields of subjective or neuropsychiatric research (e.g. pain), where consumer-grade electronics are increasingly being used^8,20,21^.

Variation across trials within a session proved useful for classifying participants as patients or malingers (see Fig. 5B). With chronic subjective tinnitus, the phantom sound percept is continuous but the perceptual qualities (the loudness, pitch, etc.) can fluctuate over time^8^. For this reason, we included the natural heterogeneity within a participant by compiling measurements performed in five separate test sessions across several weeks. Condensing the measurements into a single session would reduce the variability within participants and presumably lead to even more accurate classification accuracy. This prediction could be tested in future studies but, for the purposes of this study, our classification accuracy and measurement time represents a conservative estimate.

On the individual trial level, patients feigning tinnitus took less time to mask their tinnitus and had fewer reversals in their finger trajectory while matching their tinnitus. This result is in line with the expectation that it takes more time to achieve a precise match or masking of actual tinnitus than of feigned tinnitus. However, we opted to not include this as a feature in the classification and relied on a subset of the raw time-series data. The primary reason for this is that the duration could be easily adapted and would not necessarily be a robust, repeatable measure and more likely reflects participant engagement. By discarding this feature, our classification accuracy again represents a conservative estimate.

The study is limited in several ways. While the samples were matched in terms of age, they were relatively small, and not controlled based on familiarity with using the tablet, the time of day, or audiometry. Also, the malingering participants in the current study were not financially motivated to feign tinnitus to the same extent as actual malingers, whose financial renumeration could be larger but also condition upon making a credible claim. Because of the simplicity of the tests reported and the minimal amount of patient time required to conduct them, more robust classifiers could be developed on larger datasets through online testing. A larger study could also be validated against a true held-out dataset rather than rely on cross-validation.

Conventional subjective tinnitus assessments based on questionnaires cannot easily detect malingerers. In the broader context of tinnitus clinical research, questionnaire assessments could inflate placebo effects, underestimate treatment effects, and generally obscure the true prevalence and severity of tinnitus in the population^22–24^. While objective biomarker measurements are the gold standard and the ultimate aim for tinnitus diagnostics, automatic and quantitative assessments of biobehavioral data represent an important intermediate step. Further to this point, as studies are progressively moving to online formats, these data can be scaled up to larger cohorts and subjected to similar forms of classification analyses described here. It seems likely that automated analyses of standardized rapid tinnitus tests would inform the decision as to whether to accept or deny claims of tinnitus disability. It could also be useful for screening participants in future clinical trials related to objective biomarkers or treatment methodologies. In this way, accurate identification of participants with tinnitus and differentiation from malingerers will also help funds be distributed where they are needed most and enable research on tinnitus severity. An important next step would be to expand the type of data used by automated classification methods to include behavioral or autonomic measures that more closely reflect the psychological burden of tinnitus, rather than just the perceptual qualities of tinnitus. Reliable biomarkers for psycho-emotional tinnitus burden have yet to be defined but—once identified—could be combined with the established psychoacoustic markers described here to reach a more comprehensive, automatic, quantitative, and individual assessment of tinnitus severity.

## Methods

### Populations

**Fig. 7:**
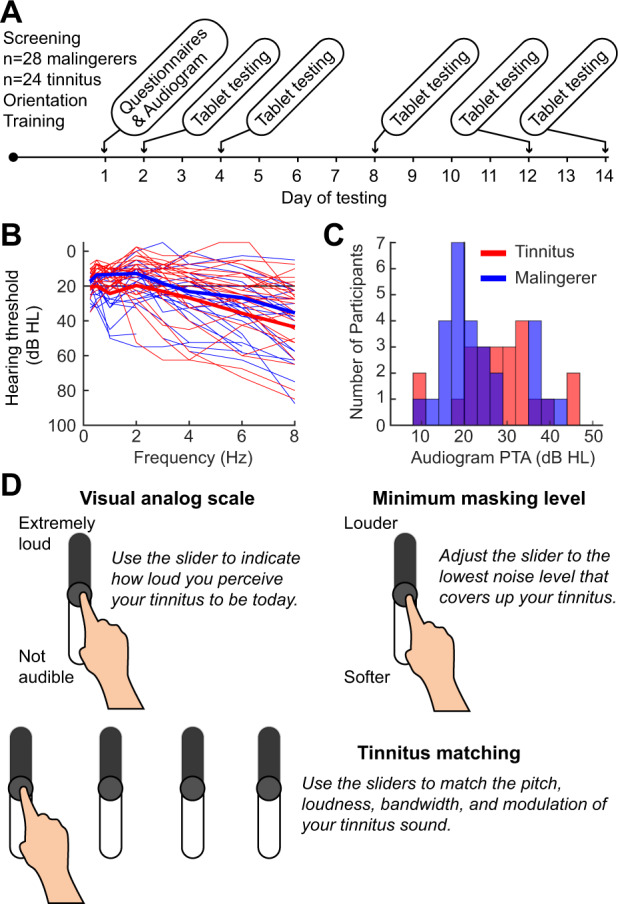
Summary of tinnitus characterization procedure. **A** Schematic of experimental design. **B**, **C** Audiograms (**A**) and pure tone average (**B**) for tinnitus patients (red) and a malingering cohort (blue), who were subsequently instructed to feign having tinnitus. **D** Graphical User Interface to characterize tinnitus loudness, minimum masking level, and to match tinnitus with visual sliders.

A population of 52 participants were included for study. The tinnitus cohort (*N* = 24) were recruited from Mass Eye and Ear, having reported bothersome, chronic subjective tinnitus for at least one year as their chief complaint, as described in preliminary form in a previous publication^8^. The malingering cohort consisted of 28 individuals who confirmed having no perception of tinnitus. This study was approved by the Massachusetts Eye and Ear and and Mass General Brigham Institutional Review Board and participants provided written informed consent to take part in the study. Subjects participated in self-directed behavioral testing in a remote, home-based setting via tablet computers and calibrated headphones, as described previously^8,25,26^ to their hearing with calibrated headphones over a period of days to behaviorally characterize their tinnitus. Figure 7A schematizes the overview of the study design, where five sessions were performed over a 2 week period following a baseline clinical assessment and audiometry. After first confirming that they did not have tinnitus, participants in the malingering groups were instructed to perform all measures as if they heard a constant phantom sound. They were first trained on what tinnitus sounded like by reading text descriptions and listening to five sample audio files of tinnitus match sounds. Matched sounds were generated from data corresponding to actual patient matches of their tinnitus precepts, and were selected to exemplify the heterogeneity in tinnitus pitch, bandwidth, and loudness found our sample^8^. After confirming that they were confident in their ability to imagine a tinnitus sound, the malingering participants were instructed to complete the tests while imagining the presence of a constant tinnitus-like sound. Apart from these additional instructions and guidance for the malingering group, all procedures were matched between the two groups.

The average ages of the patient and malingerer groups were 52.0 and 53.7, respectively. A summary of the patient demographics can be found in Table 1. Hearing (i.e. audiometric) thresholds were measured for both ears between 0.25 and 8 kHz. Figure 7B–C shows the individual and average audiograms for the two groups, respectively. All participants were financially compensated for their participation (equally between the tinnitus and malingerer group).

**Table 1:** Mean demographic for the tinnitus patient and malingerer groups.

	Tinnitus	Malingerer
Number of Participants	24	28
Age	52.0 (12.3)	53.7 (11.4)
Audiogram Pure Tone Average	28.4 (9.2)	24.0 (8.6)
Tinnitus Handicap Inventory	29.1 (12.0)	22.1 (12.0)

Standard Deviations are shown in parentheses. Note that malingers were asked to answer the THI questionnaire as if they had tinnitus.

### Tinnitus characterization

All participants performed three psychoacoustic tasks: a tinnitus visual analog scale (VAS) rating, the minimum masking level (MML) measurement, and the tinnitus acoustic matching, all of which used a slider-bar and touch screen to collect the participant’s response. For each of the five test sessions, multiple runs (i.e. repetitions) were performed for each measurement type. Each of these repetitions within a session are referred to as trials. Subjects performed all testing from home using calibrated circumaural headphones (Bose) and custom software applications developed as a Windows Store App using the Unity game engine and side-loaded onto the tablets (Microsoft Surface Pro 2).

Figure 7D depicts the participant view of the three slider tasks and their instructions. The VAS slider ranged from ’not audible’ to ’extremely loud’ (0–100). In the MML task, participants adjusted a virtual slider to control the sound level of a noise band. Their task was to identify the minimum sound level at which they no longer perceived their (imagined) tinnitus over the masking noise. Finally, participants were then asked to adjust the acoustic properties of sound delivered to one ear so as to match the sound of their real or imagined tinnitus percept. The sound was presented monaurally, to the ear where participants reported having less tinnitus, thus allowing them to compare the sound generated by the tablet software to their perceived or imagined tinnitus sound. Subjects adjusted four auditory characteristics: level (dB SL), center frequency (Hz), bandwidth (octaves), and amplitude modulation (Hz). They could adapt these characteristics with sliders on the tablet via interaction with the touchscreen, where the sound could be changed procedurally as they adjusted the sliders. The parameters used to generate the audio were sampled and stored at approximately 5 times per second (5 Hz) from the slider values. The arrangement of the sliders and the range of values the slider encoded became smaller over trials within a session. The same distance (i.e., the values covered, for instance, a change in sound level) a finger traveled on the slider in trial 1 does not necessarily correspond to the change in level in trial 10, thus encouraging subjects to attend to real-time changes in acoustic feedback as they moved their finger on the tablet surface rather than the position of the slider from previous trials.

### Feature extraction

Two approaches were used to analyze the participant’s interaction with the graphical touch sliders, corresponding to *what* was measured and *how* it was answered. The first approach focused on the response outcomes, which correspond to standard pyschoacoustic measures of tinnitus including the masking level, VAS and tinnitus matching. These standard measures are derived from the final value of the graphical slider on each trial of the experiment. A repeated measures ANOVA with the factors session (1–5) and group (patient, malingerer) was then used to determine whether there were any differences between the reported precept of the two populations, as well as changes in test-retest reliability. Participants were modeled as a random effect. The second method focused on how participants interacted with the graphical user interface and sliders over time, not just the final value of the slider. Figure 4A–B illustrates how the change in slider value over time can be recorded and stored as a time series.

### Machine learning

Classifier algorithms were constructed to distinguish between tinnitus and malingerer groups. Leave-one-subject-out (LOSO) cross-validation was used to train and evaluate the model, combining runs of slider adjustment across the various tinnitus-characterization tasks within an experimental session. Classifiers were evaluated in terms of classification accuracy (percent correct), and by the area under the curve (AUC) of the receiver operating characteristic (ROC) curve, which evaluates the diagnostic capability of a binary classifier^27^.

Classification was performed with three different types of input data and corresponding classifiers. First the outcome measurements alone were used (i.e. the final slider values) as input using (multiple) logistic regression. Second, the MML slider time series was used as input to a convolutional deep neural network (DNN) to investigate latent information stored in the slider movement. Finally, summary features were derived and pooled from the time-series data of all tasks. Feature-based classifier implementation was done in Python using scikit-learn’s (version 0.24.2) LogisticRegression and RandomForestClassifier^28^ in a leave-one-participant out fashion. The random forest classifier was run with with 100 estimators and a maximum depth of 5 trees. Features derived included the slider final value, the mean and standard deviation of the slider time-series across runs as well as the maximum velocity, and cross-correlation of the time-series across runs.

Alternatively, a DNN was applied to the raw slider time series data derived from the tinnitus masking task that did not depend on manual feature selection. Because the time-series may be a different length for each participant and each trial, only the final portion of slider time-series for each participant was used (see Fig. 4C). We determined that 15 s was long enough to capture the dynamics of finger movements as subjects adjusted the virtual slider but short enough to avoid data exclusion. This fixed duration segmentation also had the benefit of removing time spent on each trial as a latent feature (i.e. participant effort).

The DNN was implemented in pytorch 1.3.1 (https://pytorch.org/docs/1.3.1/), and used two cascaded 1D convolutional layers (18 and 9 channels respectively), followed by maxpool, batchnorm, and a fully connected layer. Eighteen input channels corresponded to the 18 trials of the MML task performed in a single session (270 s total). Each of the five sessions for each participant were evaluated separately by the DNN, but all sessions were held out for the test subject . Dropout was used during training with a probability of 10%.

To separate *how* the participant interacted with the slider from *what* the final result of the slider revealed (i.e. the MML) we subtracted the mean slider final value from the time series for each participant. This process normalizes the actual selected behavioral value so that the final value is 0 dB SL on average (Fig. 4).

### Reporting summary

Further information on research design is available in the Nature Research Reporting Summary linked to this article.

## Supplementary information

Reporting Summary

## Data Availability

The data are available from the corresponding author on reasonable request.

## Code availability

The code are available from the corresponding author on reasonable request.

## References

[1] Maes IH, Cima RF, Vlaeyen JW, Anteunis LJ, Joore MA (2013). Tinnitus: a cost study. Ear Hearing.

[2] Bhatt JM, Lin HW, Bhattacharyya N (2016). Prevalence, severity, exposures, and treatment patterns of tinnitus in the united states. JAMA Otolaryngol. Head Neck Surg..

[3] Basile C-É, Fournier P, Hutchins S, Hébert S (2013). Psychoacoustic assessment to improve tinnitus diagnosis. PloS One.

[4] Yakunina N, Nam E-C (2021). What makes tinnitus loud?. Otology Neurotology.

[5] Henry JA (2016). "Measurement” of tinnitus. Otology Neurotology.

[6] Byun JY, Yeo SG, Park MS (2010). Reliability of repeated tinnitogram as malingering test. Otolaryngol. Head Neck Surg..

[7] Henry JA, Meikle MB (2000). Psychoacoustic measures of tinnitus. J. Am. Acad. Audiology.

[8] Chen JX, Whitton JP, Parthasarathy A, Hancock KE, Polley DB (2020). Fluctuations in subjective tinnitus ratings over time: Implications for clinical research. Otology Neurotology.

[9] Hiller W, Goebel G, Rief W (1994). Reliability of self-rated tinnitus distress and association with psychological symptom patterns. Br. J. Clin. Psychol..

[10] Hoare DJ, Edmondson-Jones M, Gander PE, Hall DA (2014). Agreement and reliability of tinnitus loudness matching and pitch likeness rating. PloS One.

[11] Jerger S, Jerger J, Lewis S (1981). Pediatric speech intelligibility test. ii. effect of receptive language age and chronological age. Int. J Pediatr. Otorhinolaryngol..

[12] Henry JA (2013). Evaluating psychoacoustic measures for establishing presence of tinnitus. J. Rehab. Res. Dev.

[13] Steiger JR, Thielman EJ, Henry JA (2013). Tinnitus loudness tracking: A “Type V Békésy” pattern does not exist for pseudotinnitus. J. Am. Acad. Audiology.

[14] McMillan GP, Thielman EJ, Wypych K, Henry JA (2014). A bayesian perspective on tinnitus pitch matching. Ear Hearing.

[15] Khan AI, Shah JL, Bhat MM (2020). Coronet: a deep neural network for detection and diagnosis of covid-19 from chest x-ray images. Comput. Methods Prog. Biomed..

[16] Krizhevsky A, Sutskever I, Hinton GE (2012). Imagenet classification with deep convolutional neural networks. Adv. Neural Inf. Process. Syst.

[17] Nassif AB, Shahin I, Attili I, Azzeh M, Shaalan K (2019). Speech recognition using deep neural networks: a systematic review. IEEE access.

[18] Li JJ, Tong X (2020). Statistical hypothesis testing versus machine learning binary classification: Distinctions and guidelines. Patterns.

[19] Tolles J, Meurer WJ (2016). Logistic regression: relating patient characteristics to outcomes. JAMA.

[20] Yang YS, Ryu GW, Choi M (2019). Methodological strategies for ecological momentary assessment to evaluate mood and stress in adult patients using mobile phones: systematic review. JMIR mHealth uHealth.

[21] Henry JA, Roberts LE, Ellingson RM, Thielman EJ (2013). Computer-automated tinnitus assessment: noise-band matching, maskability, and residual inhibition. J. Am. Acad. Audiology.

[22] UK National Guideline Centre. Evidence review for psychoacoustic measures: Tinnitus: assessment and management: Evidence review I (2020). https://www.ncbi.nlm.nih.gov/books/NBK557025/. 32437112

[23] Landgrebe M (2012). Methodological aspects of clinical trials in tinnitus: a proposal for an international standard. J. Psychosomatic Res..

[24] Fava M, Evins AE, Dorer DJ, Schoenfeld DA (2003). The problem of the placebo response in clinical trials for psychiatric disorders: culprits, possible remedies, and a novel study design approach. Psychother. Psychosomatics.

[25] Whitton JP, Hancock KE, Shannon JM, Polley DB (2017). Audiomotor perceptual training enhances speech intelligibility in background noise. Curr. Biol..

[26] Whitton JP, Hancock KE, Shannon JM, Polley DB (2016). Validation of a self-administered audiometry application: an equivalence study. Laryngoscope.

[27] Fawcett T (2006). An introduction to ROC analysis. Pattern Recognit. Lett..

[28] Pedregosa F (2011). Scikit-learn: Machine learning in Python. J. Mach. Learn. Res..

