# Supplementary material for: Automatic identification of tinnitus malingering based on overt and covert behavioral responses during psychoacoustic testing
Source: NPJ Digit Med. 2022 Aug 29;5:127. doi: 10.1038/s41746-022-00675-w (PMC9424223; doi:10.1038/s41746-022-00675-w)
Supplement: Supplementary file 1 — Reporting Summary [file 41746_2022_675_MOESM1_ESM.pdf]

nature portfolio

Corresponding author(s): Dan Polley, Christopher Smalt

Last updated by author(s): Jun 8, 2022

# Reporting Summary

Nature Portfolio wishes to improve the reproducibility of the work that we publish. This form provides structure for consistency and transparency in reporting. For further information on Nature Portfolio policies, see our Editorial Policies and the Editorial Policy Checklist.

## Statistics

For all statistical analyses, confirm that the following items are present in the figure legend, table legend, main text, or Methods section.

| n/a | Confirmed | |
|---|---|---|
| ☐ | ☒ | The exact sample size (*n*) for each experimental group/condition, given as a discrete number and unit of measurement |
| ☐ | ☒ | A statement on whether measurements were taken from distinct samples or whether the same sample was measured repeatedly |
| ☐ | ☒ | The statistical test(s) used AND whether they are one- or two-sided<br>*Only common tests should be described solely by name; describe more complex techniques in the Methods section.* |
| ☒ | ☐ | A description of all covariates tested |
| ☐ | ☒ | A description of any assumptions or corrections, such as tests of normality and adjustment for multiple comparisons |
| ☐ | ☒ | A full description of the statistical parameters including central tendency (e.g. means) or other basic estimates (e.g. regression coefficient) AND variation (e.g. standard deviation) or associated estimates of uncertainty (e.g. confidence intervals) |
| ☐ | ☒ | For null hypothesis testing, the test statistic (e.g. *F*, *t*, *r*) with confidence intervals, effect sizes, degrees of freedom and *P* value noted<br>*Give P values as exact values whenever suitable.* |
| ☒ | ☐ | For Bayesian analysis, information on the choice of priors and Markov chain Monte Carlo settings |
| ☒ | ☐ | For hierarchical and complex designs, identification of the appropriate level for tests and full reporting of outcomes |
| ☒ | ☐ | Estimates of effect sizes (e.g. Cohen's *d*, Pearson's *r*), indicating how they were calculated |

*Our web collection on statistics for biologists contains articles on many of the points above.*

## Software and code

Policy information about availability of computer code

| | |
|---|---|
| Data collection | Custom code written for the Microsoft Surface Tablet platform to collect the data. This collection methodology is described in detail in previous papers (Chen et al., 2020; Whitton et al., 2017, 2016) |
| Data analysis | Feature-based classifier implementation was done in Python using scikit-learn's (version 0.24.2) LogisticRegression and RandomForestClassifier. The DNN classifier was implemented in pytorch 1.3.1. |

For manuscripts utilizing custom algorithms or software that are central to the research but not yet described in published literature, software must be made available to editors and reviewers. We strongly encourage code deposition in a community repository (e.g. GitHub). See the Nature Portfolio guidelines for submitting code & software for further information.

## Data

Policy information about availability of data

All manuscripts must include a data availability statement. This statement should provide the following information, where applicable:

- Accession codes, unique identifiers, or web links for publicly available datasets
- A description of any restrictions on data availability
- For clinical datasets or third party data, please ensure that the statement adheres to our policy

The data are available from the corresponding author on reasonable request

## Human research participants

Policy information about studies involving human research participants and Sex and Gender in Research.

| | |
|---|---|
| Reporting on sex and gender | sex and/or gender was determined based on self-report |
| Population characteristics | Adult subjects were matched based on age and clinical hearing status, to the extent possible |
| Recruitment | Tinnitus subjects were recruited via email notice to a Boston-area tinnitus patient support group. The malingering subjects were recruited through a clinical research flier for subjects who did not have tinnitus. |
| Ethics oversight | Study was approved by the Massachusetts Eye and Ear and and Mass General Brigham Institutional Review Board |

Note that full information on the approval of the study protocol must also be provided in the manuscript.

# Field-specific reporting

Please select the one below that is the best fit for your research. If you are not sure, read the appropriate sections before making your selection.

☐ Life sciences ☒ Behavioural & social sciences ☐ Ecological, evolutionary & environmental sciences

For a reference copy of the document with all sections, see nature.com/documents/nr-reporting-summary-flat.pdf

# Behavioural & social sciences study design

All studies must disclose on these points even when the disclosure is negative.

| | |
|---|---|
| Study description | The data include both quantitative and qualitative measures. |
| Research sample | Tinnitus subjects were recruited via email notice to a Boston-area tinnitus patient support group. The malingering subjects were recruited through a clinical research flier for subjects who did not have tinnitus. |
| Sampling strategy | Sample size was pre-determined since the tinnitus patient data was re-used from a previous study (Chen 2020) |
| Data collection | A tablet computer and headphones were used to collect the data. The data were collected remotely through self-directed automated testing and the investigators were not present during the time of collection. |
| Timing | For Malingerer group, the first subject enrolled on 12/11/20 Final subject completed study on 8/12/21. Data from tinnitus subjects were collected as part of an earlier study (as reported in Chen et al., 2020). |
| Data exclusions | Two subjects was excluded from the malingering group because they realized that they did have tinnitus part of the way through the study. |
| Non-participation | One subject consented but decided not to begin the study for personal reasons. |
| Randomization | Participants were age-matched between groups. |

# Reporting for specific materials, systems and methods

We require information from authors about some types of materials, experimental systems and methods used in many studies. Here, indicate whether each material, system or method listed is relevant to your study. If you are not sure if a list item applies to your research, read the appropriate section before selecting a response.

## Materials & experimental systems

| n/a | Involved in the study |
| --- | --- |
| ☒ | ☐ Antibodies |
| ☒ | ☐ Eukaryotic cell lines |
| ☒ | ☐ Palaeontology and archaeology |
| ☒ | ☐ Animals and other organisms |
| ☒ | ☐ Clinical data |
| ☒ | ☐ Dual use research of concern |

## Methods

| n/a | Involved in the study |
| --- | --- |
| ☒ | ☐ ChIP-seq |
| ☒ | ☐ Flow cytometry |
| ☒ | ☐ MRI-based neuroimaging |
